# Fungal and bacterial species richness in biodeteriorated seventeenth century Venetian manuscripts

**DOI:** 10.1038/s41598-024-57228-2

**Published:** 2024-03-25

**Authors:** Maria Stratigaki, Andrea Armirotti, Giuliana Ottonello, Sabrina Manente, Arianna Traviglia

**Affiliations:** 1 Center for Cultural Heritage Technology (CCHT), Istituto Italiano di Tecnologia, Via Torino 155, 30172 Venice, Italy; 2 Analytical Chemistry Facility, Istituto Italiano di Tecnologia, Via Morego 30, 16163 Genoa, Italy; 3 Department of Molecular Sciences and Nanosystems, Ca’ Foscari University of Venice, Via Torino 155, 30172 Venice, Italy

**Keywords:** Biological techniques, Imaging, Mass spectrometry, Microbiology techniques, Proteomic analysis, Microbiology, Microbial communities, Pathogens, Characterization and analytical techniques, Imaging techniques, Microscopy

## Abstract

Historical paper documents are susceptible to complex degradation processes, including biodeterioration, which can progressively compromise their aesthetic and structural integrity. This study analyses seventeenth century handwritten historical letters stored at the Correr Museum Library in Venice, Italy, exhibiting pronounced signs of biodeterioration. The techniques used encompassed traditional colony isolation on agar plates and proteomics analyses, employing nanoscale liquid chromatography coupled with high-resolution mass spectrometry (nano-LC–MS). Fluorescence microscopy was used for the first time in the historical paper biodeterioration context to supplement the conventional stereoscopic, optical, and scanning electron microscopic imaging techniques. This method enables the visualisation of microorganisms beyond and beneath the paper’s surface through their natural intrinsic autofluorescence in a non-invasive and non-destructive way. The results demonstrate a diverse, complex, and abundant microbiota composed of coexisting fungal and bacterial species (Ascomycota, Mucoromycota, Basidiomycota, Proteobacteria, and Actinobacteria), along with mite carcasses, insects, parasites, and possibly protists. Furthermore, this study reveals certain species that were not previously documented in the biodeterioration of historical paper, including human pathogens, such as *Histoplasma capsulatum*, *Brucella*, *Candida albicans*, and species of *Aspergillus (A. flavus, A. fumigatus, A. oryzae, A. terreus, A. niger)* known to cause infections or produce mycotoxins, posing substantial risk to both artefacts and humans.

## Introduction

Handwritten codices, documents, and letters are valuable cultural heritage materials exhibiting exquisite artisanry and possessing immense value as they preserve, convey, and communicate centuries-old written information across extended periods of time. Their paper substrates, however, suffer from natural ageing^1,2^, which significantly undermines their long-term stability. Numerous factors contribute to the degradation of paper, and these factors can be categorised according to their nature. Physicochemical factors include light, temperature, relative humidity, pH, microclimate, storage conditions, hydrolysis and oxidation, composition, raw materials, fillers and additives^3–6^. Mechanical factors cause wear and tear accumulation^7^, while biological factors arise from the colonisation of microorganisms, primarily fungi and bacteria, producing corrosive metabolites^8,9^. The degradation processes caused by these factors render paper-based writing supports fragile and vulnerable, thereby endangering their preservation for the future.

The biodeterioration of writing substrates is the result of intricate interactions at the micro- and nano-scale, leading to discoloration, foxing, embrittlement, cracking, or, ultimately, complete loss of structural integrity^10–12^. Certain fungal species, for example, produce metabolic waste that causes visible coloured stains on the substrate at a macro-level. Active metabolites, such as enzymes and acids, secreted by these microorganisms can further lead to the acid hydrolysis of paper^13^. Yeasts, members of the Fungal Kingdom, are highly dependent on aerial spread and produce pseudo-hyphae to replicate and move to the substrate^14^. Bacteria also pose a significant threat to paper degradation due to their strong metabolite products. These microorganisms can remain viable but in a dormant state for years^15,16^, and reactivate upon exposure to optimal conditions in a favourable environment. Microbial colonisation can thus thrive in extreme environments^17,18^ or conditions with elevated levels or sudden fluctuations in temperature or humidity, which accelerate the saturation of paper substrate with water, although this is not a strict requirement.

The cataloguing and mapping of fungal and bacterial diversity, facilitated by different techniques for detection and identification, lays the foundation for protecting historical paper documents^19^. This approach prevents irreversible damage over time, allowing the public to access these documents without substantial loss. However, cultivating bacterial and fungal structures using traditional in-vitro methods can be challenging due to factors such as the selectivity of the media, temperature, and time^20–22^. To address these limitations, molecular approaches offer significant benefits in detecting the species’ fingerprint^23^. Omics techniques are increasingly used in the field of cultural heritage^24,25^ to understand the biodegradation mechanisms of microbial communities and assess biodeterioration levels. Proteomics, in particular, can analyse residual trace proteomes discovered on manuscript pages, providing valuable insight into the health conditions or causes of death of the writers^26,27^.

The use of traditional imaging methods, like optical microscopy (OM) and scanning electron microscopy (SEM), to locate species on the surface of the paper fibre network is prevalent in this field. However, to improve our understanding of intricate and complex biodegradation processes^28^, it is essential to visualise microorganisms beyond and beneath this surface. Here, we introduce a novel methodology in the field of cultural heritage and historical paper biodeterioration. Our approach utilises fluorescence microscopy as a non-invasive and non-destructive imaging technique. The method relies on the natural intrinsic autofluorescence of fungal and bacterial cells, resulting from endogenous fluorophores^29^, instead of externally integrated fluorescent markers^30,31^. Although the autofluorescence of these species is well-documented^32–34^, only a few reports have investigated this possibility in the field.

This study focused on unravelling the microbial threats posed to seventeenth century historical handwritten letters, stored in the Library of the Correr Museum (Biblioteca del Museo Correr) in Venice. These letters originate from Veneto, Italy, a region renowned for its long tradition and rich history in papermaking^35^. To conduct an in-depth investigation and evaluation of the microbial presence and diversity, a synergistic approach was adopted^36–38^. To reveal and assess individual features and patterns, different visualisation methods including stereoscopic, optical, fluorescence and scanning electron microscopy, were utilised*.* The microbial contaminant species were isolated using conventional cultures. For partial identification, phenotypic procedures were followed, and comparisons were made to illustrated references of known species. The detection and identification of proteins were carried out using nano-LC–MS, a powerful tool that requires minimal sample volume^39^. The protein content was directly digested and the corresponding peptides were analysed through mass spectrometry. The results reveal a diverse microbial community consisting of both fungal and bacterial species, alongside mite carcasses, insects, parasites, and possibly protists. There are also species that have not been previously reported in historical paper biodeterioration literature. Notably, some of these species are human pathogens causing infections or producing mycotoxins. These findings provide crucial insights for the tailored and optimised development of conservation treatments and preventive strategies employing advanced materials^40,41^.

## Results and discussion

### Macroscopic and microscopic observations on fragments

**Figure 1:**
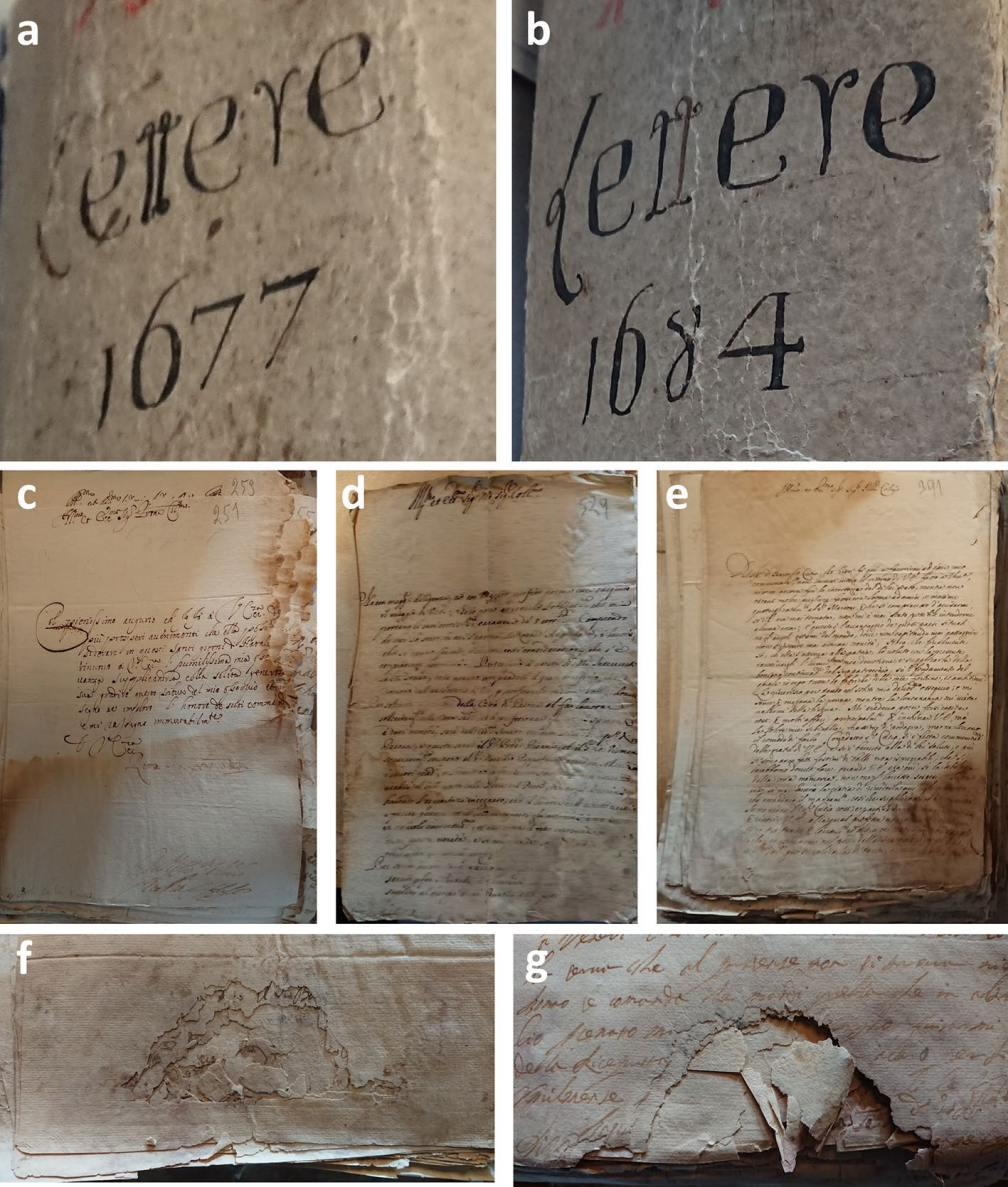
Images of historical handwritten letters dating back to 17th-century: (**a**, **b**) envelopes dating 1677 and 1684, (**c**, **d**, **e**) typical biodegradation effects which manifest as discoloration and staining, (**f**, **g**) cracking, fragmenting, and loss of mechanical and structural integrity. Images are not to scale.

A preliminary macroscopic visual assessment of the letters confirmed the extent of damage manifesting as stains, spots, foxing, tidelines, paper cracking and loss of structural integrity (Fig. 1). The deterioration was pronounced, demonstrated by the weakened paper structure and by local or widespread discoloration. The two envelopes containing a vast collection of letters dated 1677 and 1684 (Fig. 1a,b) suffered severe damage and disruption at their lower parts (Fig. 1c–e). The extent of destruction was so extensive, with multiple small fragments breaking off and detaching (Fig. 1f,g).

**Figure 2:**
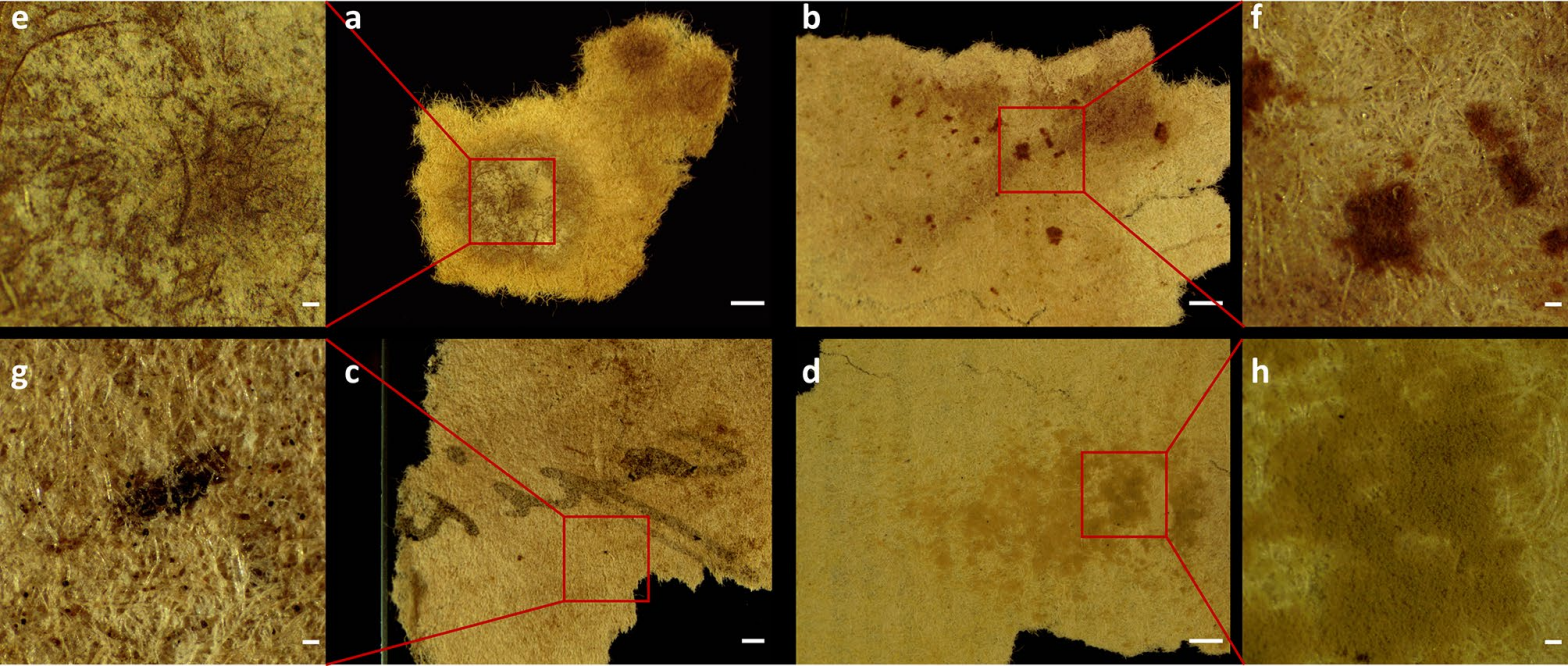
Collection of the four paper fragments: #1 (**a**, **e**), #2 (**b**, **f**), #3 (**c**, **g**), and #4 (**d**, **h**). Stereoscopic images (**a**, **b**, **c**, **d**), scale bar 1 mm. Selected areas (red squares) denote ROI under investigation, shown in detail through higher magnification imaging with the stereoscope (**e**, **f**, **g**, **h**), scale bar 100 µm.

Selected paper fragments (#1, #2, #3, #4) exhibiting stains and spots on their surface, varying in colour, shape, size, and texture were visualised using a stereoscope in reflected mode (Fig. 2). Figure 2a portrays fragment #1 with a white-yellow circular stain measuring approx. 4 mm in diameter, while Fig. 2b shows fragment #2 with several brown–red coloured spots ranging in size from 100 µm up to 1 mm. Black spots between 10 and 100 µm in size, measuring up to 1 mm, can be observed on fragment #3 (Fig. 2c), while fragment #4 is marked by orange-brown stains with a felt-like consistency that cover areas of several mm (Fig. 2d). Regions of interest (ROI) are denoted with red squares and are shown in detail through higher magnification imaging with the stereoscope (Fig. 2e–h).

**Figure 3:**
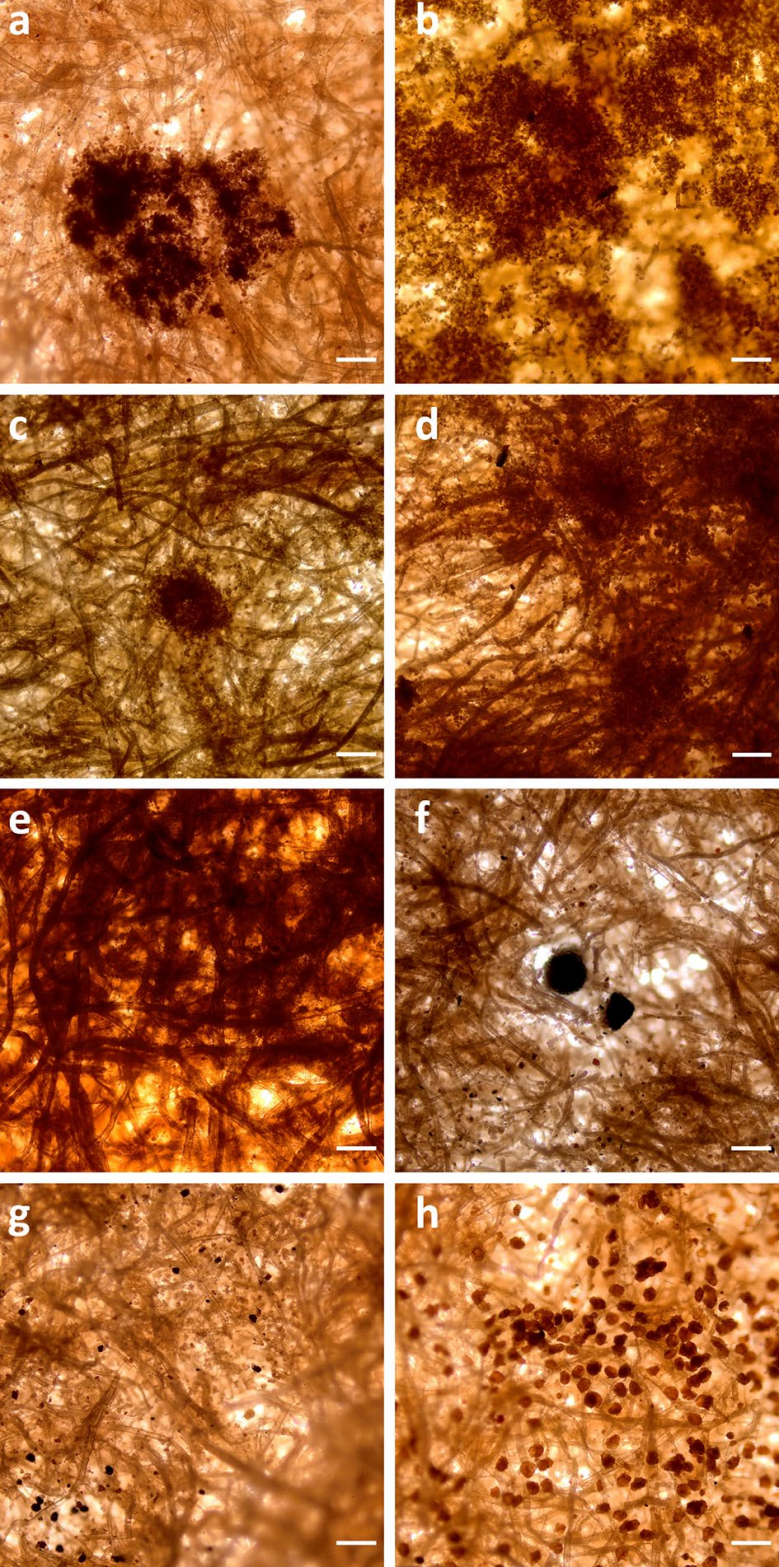
Optical microscopy images of selected ROI depicting: (**a**, **b**, **c**, **d**, **e**) spots, lesions, stains, and patches either localised covering an area of a few µm or expanding over large surface areas from several hundreds of µm up to a few mm, and (**f**, **g**, **h**) spherical deposits and spores embedded within the paper matrix. Scale bar 100 µm.

These areas were subsequently visualised using an optical microscope to attain more detailed information locally (Fig. 3). The images confirm that regions displaying distinctive characteristics at the macroscale (Fig. 2) also exhibit distinct features at the microscale. Spots, stains, lesions, and irregular patches can be localised and spread over only a few hundred µm or expand over large surface areas up to few mm (Fig. 3a–e, corresponding to fragments #1, #2, #4). To facilitate the distinction between affected and non-affected areas, microscopy images of Whatman filter paper are provided at different magnifications in Supplementary Fig. 1a–c. The paper network structure typically displays some variability in fibre distribution, pores, and channels, yet without the pronounced heterogeneity observed in the biodegraded fragments.

The optical microscopy images depicted in Fig. 3f–h correspond to fragment #3 and present black spherical deposits, measuring 95 µm (Fig. 3f). The deposits bear resemblance to the characteristic melanised perithecia of *Chaetomium globosum*^42^, a fungus with high cellulolytic activity^43^, which frequently colonises and decomposes paper materials. Figure 3g,h illustrates pigmented black and brown spores, measuring about 5–40 µm in size. These structures are fruiting bodies that produce spores^15^, and are anchored and embedded deeply within the paper matrix. Their survival and colonisation mechanism^44,45^ relies on the material degradation, which starts from the surface and penetrates deep into the layers^21^.

**Figure 4:**
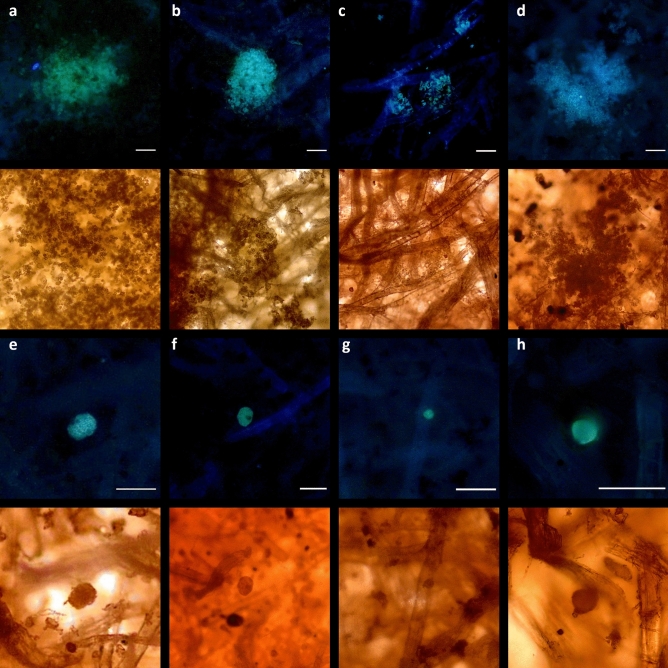
Optical microscopy images under fluorescence (top) and brightfield (bottom) modes showing autofluorescent microorganisms within the paper fibre network: (**a**, **b**, **c**, **d**) spherical 3–5 µm structures entangled into the network, forming clusters covering regions of 50–200 µm, and (**e**, **f**, **g**, **h**) individual 10–30 µm spherical spores attached to single fibres. Scale bar 40 µm.

Biological structures were visualised using the optical microscope’s fluorescence illumination system. Figure 4 depicts microorganisms in both brightfield and fluorescence modes, enabling clear distinction of their autofluorescent structure from the dark background. Spherical structures measuring 3–5 µm are entrapped and entangled in the network, forming clusters covering regions of 50–200 µm (Fig. 4a–d). Meanwhile, individual spherical spores of around 10–30 µm in diameter appear to be attached to a single fibre (Fig. 4e–h).

**Figure 5:**
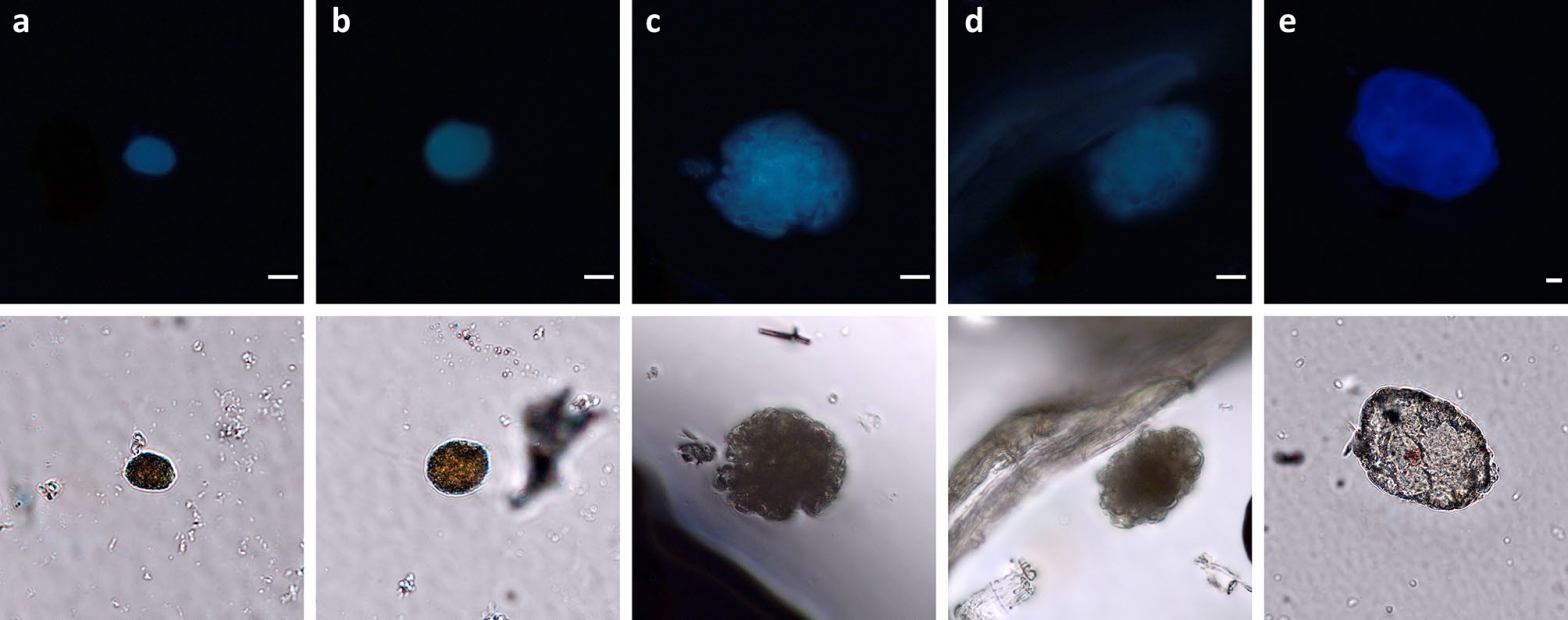
Optical microscopy images under fluorescence (top) and brightfield (bottom) modes showing autofluorescent cellular structures retrieved during imaging of microscope slides with adhesive tape pressed over the letters: (**a**, **b**) 10–20 µm with a spherical morphology, (**b**, **c**) 25–45 µm with signs of wear and tear, and (**e**) 70–100 µm with characteristic regions. Scale bar 10 µm.

**Figure 6:**
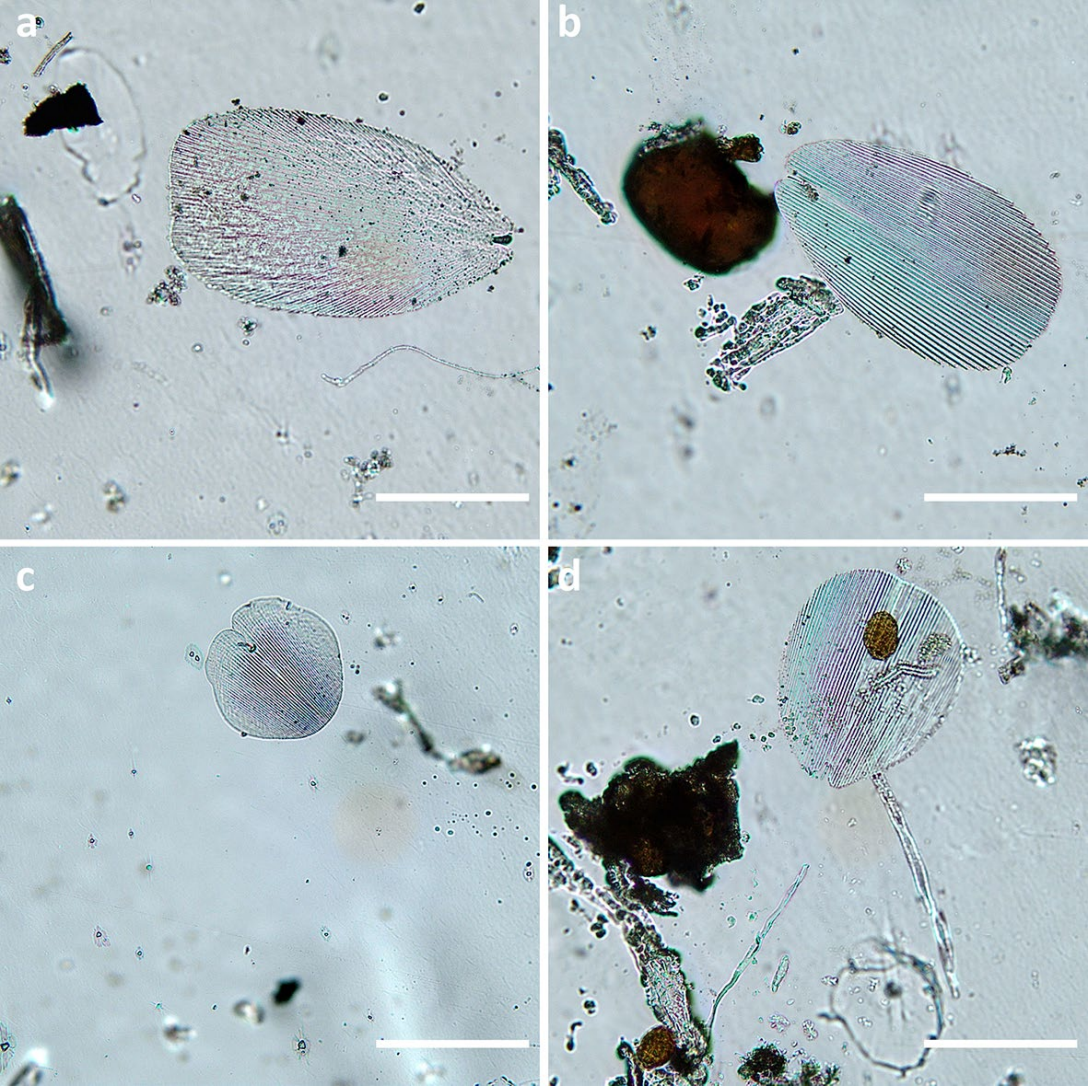
Optical microscopy images retrieved during imaging of microscope slides with adhesive tape pressed over the letters showing: (**a**, **b**, **c**, **d**) scales of the *Lepismatidae* silverfish. Scale bar 100 µm.

Autofluorescent cellular structures were detected by visualising tape pieces fixed onto microscope slides. Certain structures present a smooth, intact, spherical morphology, ranging between 10 and 20 µm (Fig. 5a,b), which can be compared to those found in a deteriorated thirteenth century Italian manuscript^23^. Other structures, measuring 25–45 µm, demonstrate signs of wear and tear (Fig. 5c,d), while all appear to be desiccated. Structures measuring 70–100 µm, sharing similarities to protists^46^, were captured (Fig. 5e). Furthermore, scales of the *Lepismatidae* silverfish^15^, now identified as *Thermobia domestica,* were observed (Fig. 6a–d). This ancient insect group can digest crystalline cellulose without microbial assistance^47^, which can potentially explain the adjacent perforations on the paper substrate.

### Imaging of fragments with scanning electron microscopy

**Figure 7:**
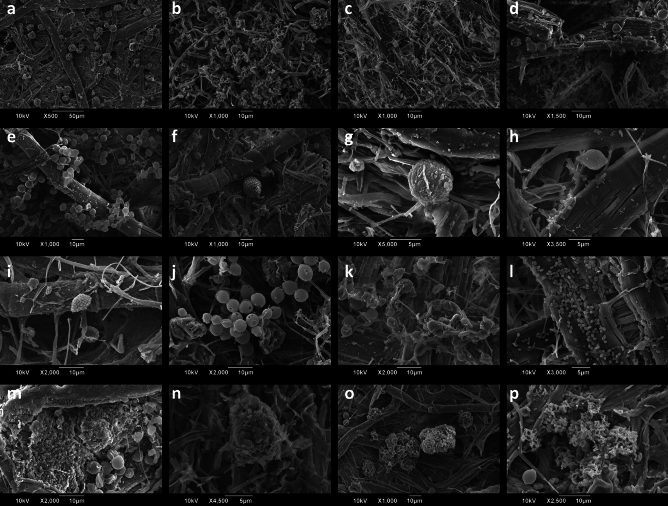
SEM micrographs showing the state of the fibre network and the different biological structures attached onto the paper fibres: (**a**, **b**, **c**) pronounced deterioration with various species and fungal hyphae infection to such an extent that the substrate is no longer visible, (**d**) torn and damaged fibres, (**e**) intact fibres with attached smooth spherical conidia of 5–6 µm in size, (**f**, **g**, **h**, **i**) various fungal structures, presumably ornamented spores: (**f**, **g**) spherical structures of 7–16 µm in diameter, (**h**, **i**) elongated and irregular with rough surfaces and length around 7–10 µm, (**j**) 5 µm spherical fungal cells, presumably belonging to *Saccharomyces cerevisiae* given the characteristic bud-scars they display on their surface, (**k**) yeast forming pseudo-hyphae, with typical elongated 5–6 µm cells with round-to-oval shape, presumably belonging to *Candida albicans*, (**l**) individual coccoid bacteria approx. 1 µm long, (**m**) bacteria next to smooth spherical 5 µm fungal conidia covering regions of more than 50 µm, (**n**) bacteria forming an agglomerate bigger than 10 µm, (**o**) 1 µm long bacteria forming agglomerate of 30 µm, (**p**) big bacterial agglomerate covering regions up to 40 µm. Scale bars and magnifications as indicated individually.

SEM images were captured to enhance visual clarity of the paper substrate and to observe *in-loco* any fungal or bacterial structures and their interactions with the fibre network. The findings confirm the pronounced biocontamination of the fragments (Fig. 7a,b). Figure 7c demonstrates the advanced fungal hyphae manifestation and entanglement with the substrate, rendering the paper invisible. Fungal hyphae pose severe risks of mechanical damage as they deeply penetrate the cellulose fibrils and fragment them^16^. The paper fibres provide physical anchorage to the fungal mycelium, resulting in the formation of a broad-mesh coating on the fibres. Fungal hyphae can move and expand extensively throughout the cellulose matrix via the mycelial network and can release coloured metabolic substances between the fibres^15^, resulting in pronounced aesthetical damage. Apparently, most of the paper fibres visible below the biocontamination are compromised, with tears, scratches, cracks, and splits along their length (Fig. 7d). Only a few fibres have remained intact despite the attached biological species. In this case, the smooth 5–6 µm spherical conidia are expanding the organism, as exemplarily shown in Fig. 7e. Figure 7f–i show the variation in fungal species with verrucose surfaces, each with different morphologies: whilst (f, g) are spherical, (h, i) are more irregular, and elongated. Figure 7j depicts spherical cells, measuring 5 µm, presumably belonging to *Saccharomyces cerevisiae*, based on the distinctive bud-scars evident on their surface^48^. Yeast presence has been observed (Fig. 7k), showing typical elongated cells with round-to-oval shape measuring 5–6 µm. These structures presumably belong to *Candida albicans*^49^, and appear alongside pseudo-hyphae, rendering removal from the substrate exceptionally difficult.

Bacterial presence was confirmed in fragments #1, #3, and #4, using SEM imaging (Fig. 7l–p). Singular coccoid bacteria measuring around 1 µm in length were found on degraded fibres (Fig. 7l), near hyphae-infected regions in proximity to smooth 5 µm spherical fungal conidia covering areas over 50 µm (Fig. 7m). Figure 7n portrays a 10 µm sticky and cohesive extracellular polymeric substance (EPS). EPS is a bacterial metabolic product which facilitates inter-communication between bacteria and contributes to their cohesive structure^36,50^. A massive 30 µm conglomerate, composed of 1 µm bacteria, located adjacent to collapsed fungal spores is shown in Fig. 7o, or with a more widespread coverage of up to 40 µm, as seen in Fig. 7p.

Bacterial adhesion typically exceeds that of fungi, compromising the longevity of paper substrates^50^. Upon the EPS excretion, a matrix is formed, into which the microorganisms become embedded^16^. This amplifies the biodeterioration process and simultaneously provides greater resistance to the microorganisms over biocidal compounds and treatments. In contrast, fungi possess exceptional adaptability to dynamic and evolving environments^51^, including those lacking nutrients and water, surpassing that of bacteria. They readily attack and invade diverse materials and translocate nutrients. Most fungi secrete extracellular adhesive enzymes into the paper substrate through their formed mycelium^9,42^. *Aspergillus* and *Penicillium* species can have a significant impact on cultural heritage materials due to their potency in producing and secreting extracellular acids and enzymes^36^.

**Figure 8:**
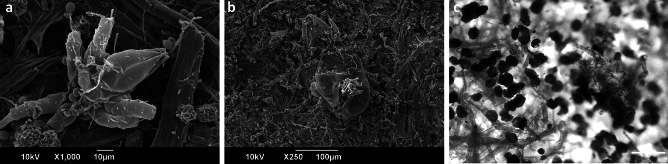
SEM and OM micrographs showing the structures of mite carcasses found in fragment #3: (**a**) mite 100 µm in size, SEM, scale bar 10 µm, (**b**) mite 250 µm in size, SEM, scale bar 100 µm, (**c**) mite 250 µm in size, OM, scale bar 100 µm.

The SEM visualisation of the samples provided evidence of two mite carcasses present on fragment #3 (Fig. 8a,b). The mite observed in Fig. 8a measures approximately 100 µm and can be associated with the Bdellid family, likely a *Bdella* species, distinguished by the elongated, snout-like gnathosoma and the pedipalps bearing two long terminal setae. The mite illustrated in Fig. 8b measures approximately 250 µm and may be attributed to *Tydeus* genus of mites, which are part of the Tydeidae family and are primarily fungivorous. Both species are predatory and are known to thrive in habitats such as soil-litter, plant leaves, moss and grasslands^52^. Optical microscopy observations also revealed the presence of a 250 µm insect in fragment #3, as shown in Fig. 8c. Dust mites of comparable dimensions were documented in biodeteriorated nineteenth century papers from Venice^37,53^, whereas other small biological structures were also observed in paper-based artefacts^54^. It should be noted that fungal and bacterial conidia attached to the mite’s hair (Fig. 8a) facilitate their dispersion and promote biodiversity by exploiting the mite as a carrier. In fact, a number of saprophytic fungi transport their spores by using the bodies of insects or mites, thereby rendering Arthropoda as their vectors^55^. Certain fungal species may also acquire nutrients to establish microbial colonisation from supplementary sources, such as dust deposits, organic residues, impurities, or dead material^15^, instead of solely relying on the paper substrate they are colonising.

The pronounced biocontamination recorded close to the exuvial remains of the three biological structures shown in Fig. 8, suggests the presence of a complex and diverse microbial population present within the paper fibre network. Notably, the mite carcass (Fig. 8a) and the bacterial conglomerate located adjacent to collapsed fungal spores (Fig. 7o) are localised views of the same area of fragment #3 (Supplementary Fig. 2a). To emphasise the amplified biodiversity, Supplementary Fig. 2b provides a stereoscopic depiction of a parasite discovered among the letters. The parasite, potentially classified as a member of *Trematoda*, inhabits freshwater environments such as rivers and streams where its host organisms (typically carnivorous mammals) reside.

### Culture-dependent analysis of species richness in paper fragments

**Figure 9:**
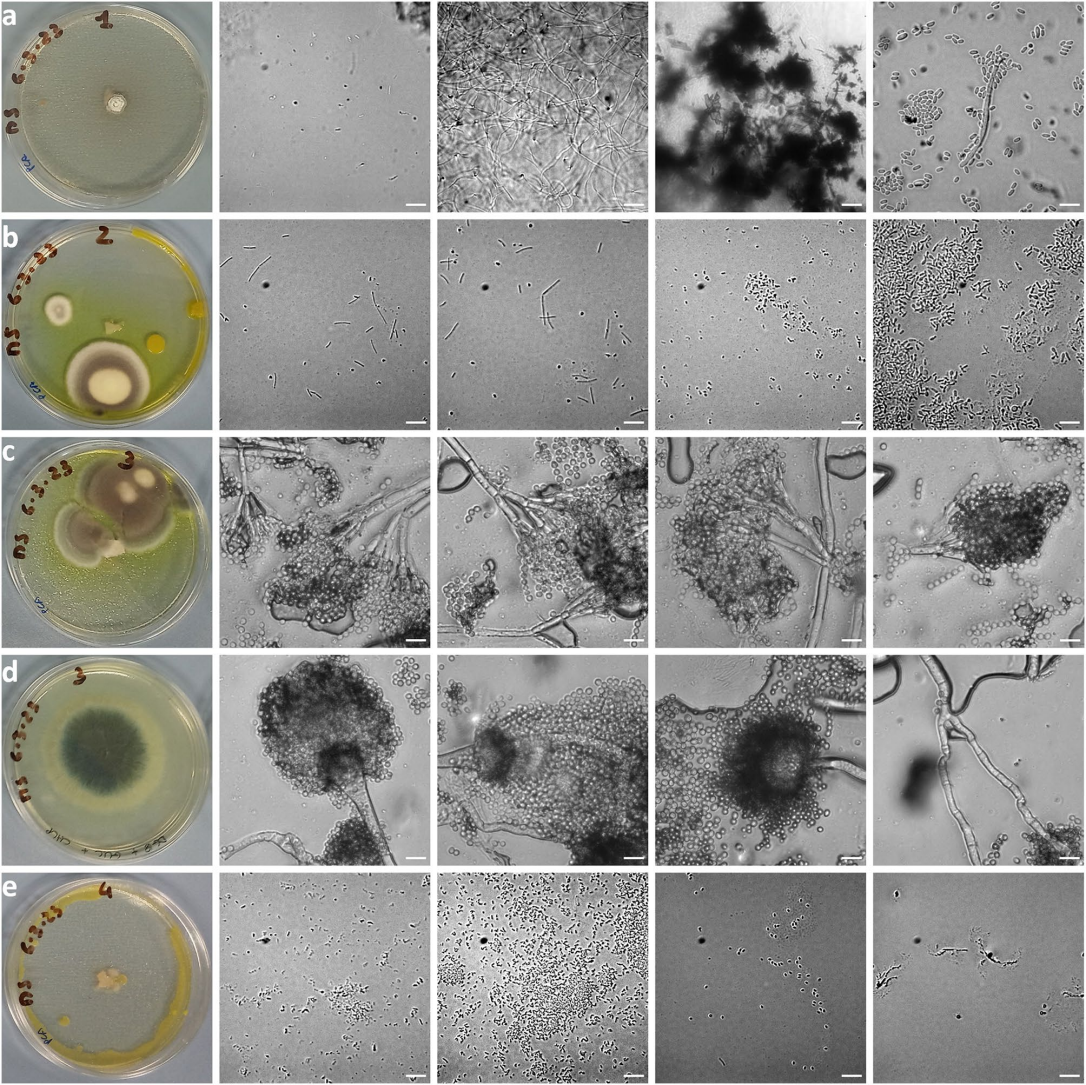
Selected images of Petri dishes after inoculation of the fragments on agar plates showing the morphology of each macro-colony captured at different days: (**a**) sample 1, PCA, day 30, (**b**) sample 2, PCA, day 15, (**c**) sample 3, PCA, day 24, (**d**) sample 3, DG18, day 15, (**e**) sample 4, PCA, day 15. Selected optical microscopy images of micro-colonies on glass slides showing the presence of fungi and/or bacteria are also provided. Scale bar 10 µm.

Four small paper fragments (#1, #2, #3, #4), along with Whatman filter paper pieces, were inoculated in different media and monitored for 30 days. Selected images showing the macro-colony morphology and the individual micro-colonies on different days are presented in Fig. 9a–e. The growth evolution of the microorganisms over time is illustrated in Supplementary Fig. 3. Information on the agar media used is provided in section “Materials and methods”. The absence of any contamination on the control plates (#0) throughout the entire duration of the experiment ensures the sterility of the biological procedure. Supplementary Fig. 3a revealed the presence of fungal and/or bacterial colonies in all four samples of the PCA media. PCA is a non-selective, general-purpose medium, recommended for the detection of live, aerobic bacteria. One fungal colony was observed in #3 for the DG18 media (Supplementary Fig. 3b). DG18 is suitable for samples with low water activity and is recommended for the selective isolation of viable osmophilic yeasts and xerophilic moulds. The included antibiotic provides additional selectivity against bacterial growth. No growth was observed for the MEA media (Supplementary Fig. 3c). MEA is a general medium recommended for the isolation of fungi. These findings demonstrate the ability of certain species to grow and proliferate under certain circumstances, as well as their resilience to nutrient-deprived environments and prolonged periods.

Figure 9a shows macro-colony #1 in PCA (day 30) and micro-colony characteristics (day 32). A hard shell was evident macroscopically and observation with optical microscopy revealed a compact and dense hypha with small, thin, pulled-out, curved branches. Such a symbiotic structure, encasing both fungi and bacteria with a cluster of hyphae, cocci, diplococci, bacilli, and streptobacilli present, was observed. This hard-shell protective structure appears as a linear hyphal aggregate of mycelial cords^56^, forming building blocks. It is characterised by a white waterproof periphery and melanised dark red-to-black coloured centre. This type of structure is typically found in symbiotic/pathogenic fungi, which serve as a source of inoculum for colonizable substrates. Thus, a symbiotic relationship between different biological species may be critical for the paper substrate, encouraging the establishment and proliferation of multiple biodeteriogenic organisms. To better understand the level of symbiosis, two recultures of the fungal and bacterial species were prepared in PCA and MEA media (Supplementary Fig. 4a). Macroscopically, the new colonies formed on the PCA culture medium resemble the primary colony of #1 in PCA (Fig. 9a). Based on morphological characteristics it is a colony belonging to *Streptomyces,* the largest genus of the Actinomycetota. *Streptomyces* comprises Gram-positive, aerobic, multicellular, filamentous bacteria capable of producing vegetative hyphae with branches. Ultimately, they can create a complex substrate akin to a fungal mycelium, with proliferation driven by dispersion of spores in a quiescent bacterial state^57^. Further optical microscopy analysis revealed a very thin, branched colonisation hyphae forming a net. Melanised, complex structures were also observed, possibly attributed to agglomerates of (pseudo) hyphae as sclerotia-like structures of fungi, typical in some strains of the *Streptomyces* species^58^. The MEA culture medium (Supplementary Fig. 4a) exhibited a large fungal colony macroscopically despite the fact that the primary culture of #1 in MEA medium did not produce any (Supplementary Fig. 3c). Microscopic imaging also captured conidia and elongated yeast cells of approx. 3–5 µm in length.

Figure 9b shows macro-colony #2 in PCA (day 15) and micro-colony characteristics (day 24). The presence of bacteria (yellow round colony) was apparent. However, the yellow-coloured halo in the agar plate was a product of a fungal colony, attributed to the genus *Penicillium* (more illustrations and details are provided in Fig. 9c). Microscopic analysis revealed the presence of living, single-cell bacteria. Some had a racket-like shape, instead of being elongated, indicating highly resistant, spore-producing organisms. In addition, small chains of 2–3 cocci, diplococci, and long bacilli, likely streptobacilli, were present. Three recultures of the bacterial species were prepared in PCA media (Supplementary Fig. 4b).

Figure 9c shows macro-colony #3 in PCA (day 24) and micro-colony characteristics (day 17). The colony is attributed to the fungal *Penicillium* genus (same as PCA #2) and does not contain any bacterial species. The macro-morphology characteristics show that the colony develops into a velvety and floccose texture with rapid growth, attaining a diameter of 4–5 cm. It produces pale yellow to green conidia and becomes darker with time, allowing it to be identified as *P. chrysogenum (*reassigned its original name, *P. rubens)*^59,60^. Indeed, the micro-morphology suggests hyaline quarter-verticillated smooth walled conidiophores, short flask-shaped thickened walled phialides, and globose and smooth conidia. The yellow pigmented halo and the exudates suggest the production of secondary metabolites, indicative of a well-conditioned colony.

Figure 9d shows macro-colony #3 in DG18 (day 15) and micro-colony characteristics (day 15). The circular colonisation hypha is of fungal origin, whereas bacterial species were not detected. The colony is assigned to *A. fumigatus* based on its macroscopic characteristics (dense, velvety, and dark green colony with aerial conidiophores) as well as its microscopic features (globose/clavate conidial heads, predominantly columnar, uniseriate phialides with short necks measuring roughly 1–3 µm in diameter, smooth to finely roughened conidia). The presence of septate hyphae divided into compartments with smooth walls and anastomosis visible is also detected in the images.

Figure 9e shows macro-colony #4 in PCA (day 15) and micro-colony characteristics (day 24). Macroscopic observations suggest the growth of bacterial colonies in different regions with distinct colours ranging from white and pink around the fragment, to yellow towards its periphery. With the use of optical microscopy, a range of bacteria, *i.e.*, cocci, micro-cocci, and diplococci, forming small chains, were observed, while no fungal species were detected. Four recultures of the bacteria species were prepared in PCA media (Supplementary Fig. 4c).

The macroscopic features of the colonies in PCA from the recultures for #1, #2, and #4 (Supplementary Fig. 4) suggest the presence of diverse bacterial strains, species, or genera. Evidently, the biodiversity unravelled within these recultures is immense. Differences in shape and colour can be observed, yet identification of a colony based solely on these macroscopic characteristics is not reliable, particularly due to the use of non-selective PCA culture media. Indeed, this generic medium promoted the growth of bacterial species, which are detectable via SEM imaging, but also exhibit notable survival and adaptive capabilities. It is important to note that these microorganisms demonstrate remarkable resilience and a significant capacity for proliferation. As a result, they present a significant risk to both artefacts and humans.

### Culture-independent analysis of species richness in paper fragments, cotton swabs, and agar plate colonies

**Figure 10:**
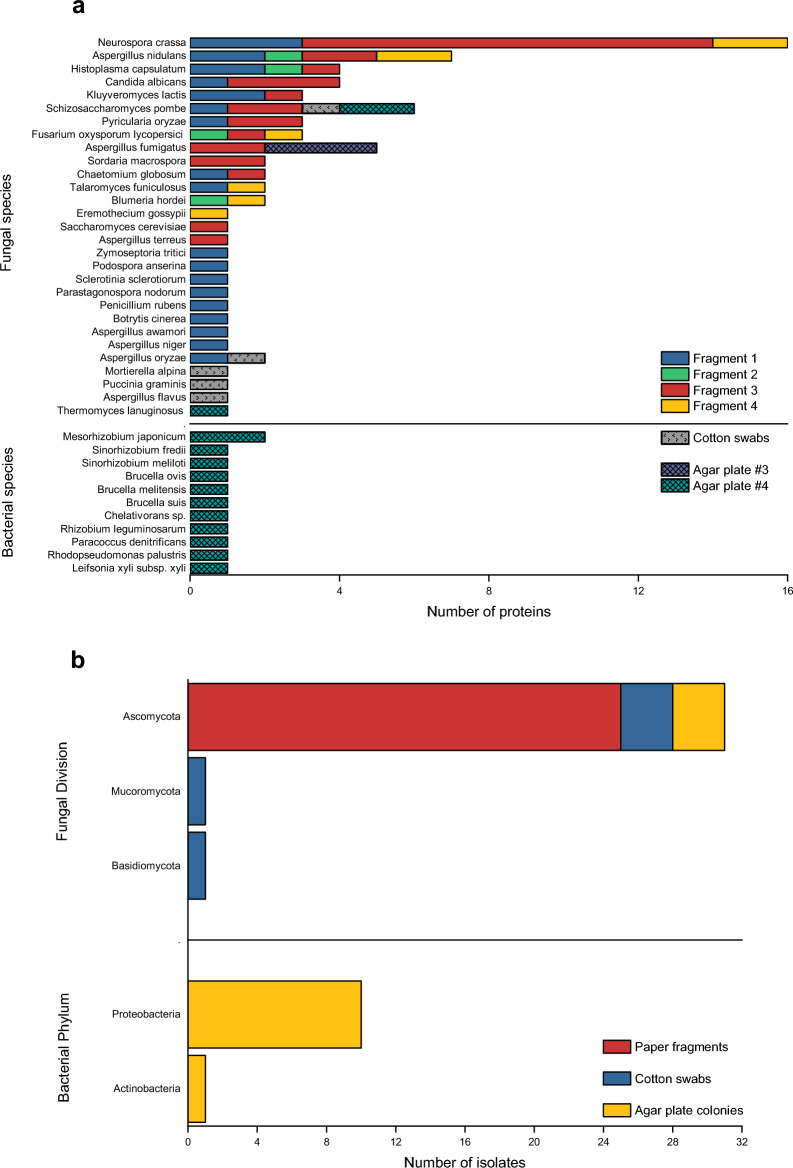
(**a**) Distribution of the fungal and bacterial species represented by the number of proteins according to the nano-LC–MS digestion and analysis of the paper fragments, cotton swabs, and agar plate colonies, and (**b**) relative abundance of isolates at the level of bacterial phylum and fungal division.

Table 1 presents the fungal and bacterial species identified from the culture-independent analysis conducted on paper fragments, cotton swabs, and agar plate colonies. Figure 10a illustrates the distribution of fungal and bacterial species based on the number of proteins, while Fig. 10b displays the relative abundance of isolates at the bacterial phylum and fungal division levels.

**Table 1:** List of fungal and bacterial species obtained from the culture-independent analysis of paper fragments, cotton swabs, and agar plate colonies.

	Paper fragments	Cotton swabs	Agar plate colonies
Fungal species			
*Neurospora crassa*	✓		
*Aspergillus nidulans*	✓		
*Aspergillus oryzae*	✓	✓	
*Aspergillus niger*	✓		
*Aspergillus awamori*	✓		
*Aspergillus terreus*	✓		
*Aspergillus fumigatus*	✓		✓
*Penicillium rubens*	✓		
*Chaetomium globosum*	✓		
*Botrytis cinerea*	✓		
*Fusarium oxysporum lycopersici*	✓		
*Talaromyces funiculosus*	✓		
*Parastagonospora nodorum*	✓		
*Sclerotinia sclerotiorum*	✓		
*Zymoseptoria tritici*	✓		
*Pyricularia oryzae*	✓		
*Blumeria hordei*	✓		
*Eremothecium gossypii*	✓		
*Candida albicans*	✓		
*Saccharomyces cerevisiae*	✓		
*Schizosaccharomyces pombe*	✓	✓	✓
*Kluyveromyces lactis*	✓		
*Sordaria macrospora*	✓		
*Podospora anserina*	✓		
*Histoplasma capsulatum*	✓		
*Mortierella alpina*		✓	
*Puccinia graminis*		✓	
*Aspergillus flavus*		✓	
*Thermomyces lanuginosus*			✓
Bacterial species			
* Mesorhizobium japonicum*			✓
* Sinorhizobium fredii*			✓
* Sinorhizobium meliloti*			✓
* Brucella ovis*			✓
* Brucella melitensis*			✓
* Brucella suis*			✓
* Chelativorans sp.*			✓
* Rhizobium leguminosarum*			✓
* Paracoccus denitrificans*			✓
*Rhodopseudomonas palustris*			✓
* Leifsonia xyli subsp. xyli*			✓

### Paper fragments

Upon searching the obtained raw mass spectral data against reference databases of bacteria and fungi (filamentous fungi and yeast) proteomes, a notable variability was observed among the four samples (#1, #2, #3, #4). A total of 25 fungal species were retrieved (Table 1, Fig. 10a), all of which belong to *Ascomycota* (Fig. 10b), one of the five phyla of the Fungal Kingdom and the most relevant to cultural heritage biodeterioration^14,51^. It is important to note that the presence of these fungi can be detrimental to the longevity of paper documents. There was no evidence of bacterial species (Table 1), attributed to a low bacterial protein load within the sensitivity limits of the analysis, as well as the fact that the samples were miniscule. Fragments #1 and #3 showed the highest number of fungal species, while fragments #2 and #4 exhibited the lowest number (Fig. 10a).

*Neurospora crassa* (#1, #3, #4), a type of filamentous fungi known for producing orange or brown spots on paper substrates^61^, had the highest number of proteins among the fungal species.

*Aspergillus nidulans,* formerly known as *Emericella nidulans*^62^, was detected in all four samples (#1, #2, #3, #4). *A. nidulans* is a species known to be involved in the biodeterioration of cultural heritage. This fungal species can infect various types of artwork objects^63^, including paper-based collections, archival and library materials^64^. It is known to produce melanins^61^ and has been identified on purple-brown stains distributed throughout a sixteenth century book, displaying migratory behaviour by repeatedly spreading through the paper pages^65^.

Sample #1 included the *Aspergillus* species *A. oryzae, A. niger, and A. awamori. A. oryzae* is an aerobic filamentous fungus that can cause foxing or produce yellow-green stains during paper decomposition^66^. It was previously isolated from a nineteenth century book^20^. *A. niger* is a filamentous fungus that is both xerotolerant and thermotolerant. It produces degrading enzymes and melanised dark brown or black staining^67^, which can lead to destruction of paper and severe loss of folding endurance due to acidic metabolites that change the pH of the paper substrate^63^. *A. niger* has been widely demonstrated to be present on paper materials^68^, and is commonly found in archival, museum, and library environments^50,69^. *A. awamori* is a fungus that produces black conidia and is known to colonize paper^15^.

Sample #3 contained *Aspergillus terreus* and *Aspergillus fumigatus. A. terreus* is an opportunistic saprotrophic fungus that produces conidia and is often found in soil. It is known for its brown and yellow pigmentation^61^, and was documented in a thirteenth century Italian manuscript^23^. The fungus secretes acidic compounds that alter the pH of the paper substrate^63^. *A. fumigatus* is a biodeteriorating agent that can be found in almost all cultural heritage materials^63^. It is also present in indoor air in archives^64,70^ and has the ability to colonise library materials, such as paper, by penetrating and decomposing the cellulose fibres^71^. *A. fumigatus* is known for its strong pH-dependent colouration properties and production of biologically active metabolites^72^, along with several melanins. This process leads to the development of brown-black and pink spots, as well as yellow and reddish-brown foxing^61^. *A*. *fumigatus* is the leading cause of human invasive aspergillosis^73^﻿﻿.

*Penicillium rubens* (#1) is a microbial colonizer of paper^15^ and was the first species known to produce penicillin^59^. It has been found in biodeteriorated nineteenth and twentieth century historical paper documents^21^. *Chaetomium globosum* (#1, #3) is a fungus that causes discoloration and biodeterioration of paper by producing melanins^61^, resulting in dark brown and grey-black spots^43^. It has been detected in the air of archives and libraries^38^, as well as in a nineteenth century book^20^. It is considered a water damage mould due to its requirement for high levels of water activity. *Botrytis cinerea* (#1) is a necrotrophic fungus that affects many plant species and crops worldwide. It is also involved in the biodeterioration of archival and library materials^71^. The fungus has been found as a contaminant on the degraded pages of a sixteenth century book^65^. *Fusarium oxysporum* f. sp. *lycopersici* (#2, #3, #4) is a fungal plant pathogen that is commonly found in biodeteriorated materials in archives and libraries^71^. It is a common cause of pink-purple stains on paper artworks^61,74^. *Talaromyces funiculosus*, formerly known as *Penicillium funiculosum*^62^, was found in two samples (#1, #4). It is a fungus that causes paper discoloration, resulting in foxing^61^, or displaying various shades of pink, orange, and red^71^.

The analysis confirmed the presence of species that had not been previously detected in biodeteriorated paper substrates. These species may have originated from contamination during the manufacturing process of the paper substrates^3^ or from subsequent handling^75^. Several parasitic fungi that infect plants have been identified, including *Parastagonospora nodorum* (#1), *Sclerotinia sclerotiorum* (#1), *Zymoseptoria tritici* (#1), *Pyricularia oryzae* (#1, #3), *Blumeria hordei* (#2, #4) and *Eremothecium gossypii* (#4). Various yeast species have been detected, including opportunistic and pathogenic *Candida albicans* (#1, #3), *Saccharomyces cerevisiae* (#3), *Schizosaccharomyces pombe* (#1, #3), and *Kluyveromyces lactis* (#1, #3). *Sordaria macrospora* (#3) and *Podospora anserina* (#1), two types of fungi typically found in mammalian dung^14^, were detected. *Histoplasma capsulatum* was found in three of the samples (#1, #2, #3). *H. capsulatum* is a fungus that is widely distributed in the environment and is the most prevalent endemic fungus worldwide^76^. It is a primary pathogen that can cause acute pulmonary disease and chronic histoplasmosis, posing a significant threat to human health^77^.

### Cotton swabs

The analysis of the sterile cotton swab tips retrieved five fungal species (Fig. 10a), including *Aspergillus oryzae* and *Schizosaccharomyces pombe,* which were previously identified in the fragments (Table 1). In addition, *Mortierella alpina*, a soil fungus of the *Mucoromycota* division, and *Puccinia graminis*, a fungus responsible for causing cereal crop disease^48^ of the *Basidiomycota* division, were identified (Fig. 10b).

*Aspergillus flavus* is a fungus with xerophilic and saprotrophic properties that has been detected. It typically grows on a variety of plants and grains^61^ and has been associated with the biodeterioration of paper^64^, causing yellow, green, or orange discoloration^38^. A. flavus is a highly toxic fungus that produces spores in large quantities^72^. It is an opportunistic pathogen in both humans and animals, and inhalation of fungal spores containing high levels of toxins can render the fungus a potent carcinogen^48^. *A. flavus* is the second cause of human invasive aspergillosis, after *A. fumigatus*^73^.

### Pristine and recultured colonies on agar plates

The analysis of pristine cultures in PCA and DG18 media, along with recultures in PCA media, revealed the presence of both fungal and bacterial species (Table 1, Fig. 10a). The bacterial community (agar plate #4) consisted of two phyla. The most prevalent phylum was Proteobacteria, and specifically, the class Alphaproteobacteria, followed by Actinomycetota or Actinobacteria (Fig. 10b).

Alphaproteobacteria respond to Gram-negative oligotrophic bacteria, which are capable of surviving in low-nutrient environments. They were represented by *Mesorhizobium japonicum*, *Sinorhizobium* (*S. meliloti, S. fredii*), *Brucella* (*B. ovis, B. melitensis, B. suis*), *Chelativorans sp.*, *Rhizobium leguminosarum*, *Paracoccus denitrificans*, and *Rhodopseudomonas palustris*. It is worth noting that *B. melitensis* and *B. suis* are responsible for human brucellosis. The species *M. japonicum*, *S. meliloti*, S. *fredii*, *Chelativorans sp*., and *R. leguminosarum* belong to Rhizobia, a group of soil bacteria that exhibit metabolic plasticity. Rhizobia form nitrogen-fixing symbioses, which is an ancient function that originated in the Archean period. Therefore, they serve as an actual example of mutualism. They are highly heterogeneous, with a high number of paralogous genes, the majority of which are ancient duplications. Interestingly, *M. japonicum* belongs to the same branch as the mammalian pathogen *Brucella*^22^. Conversely, Actinobacteria is a diverse phylum of Gram-positive bacteria, representing one of the largest taxonomic units in the bacterial domain. They inhabit soil, marine systems, and freshwater environments. *Leifsonia xyli subsp. xyli* belongs to the phylum Actinobacteria and is a fastidious plant-commensal bacterial pathogen^78^. While historical paper literature has referenced the bacterial phyla Proteobacteria and Actinobacteria^4,21,28,79^, none of these distinct species have been documented yet. The fungal community (Table 1, Fig. 10a) comprised of *Aspergillus fumigatus* (agar plate #3)*, Schizosaccharomyces pombe* (agar plate #4), and *Thermomyces lanuginosus* (agar plate #4). *T. lanuginosus* is a thermophilic fungus found worldwide that can withstand a range of environmental stressors, such as nutrient limitation and heat shock. It decomposes plant materials and grows commensally with cellulolytic fungi, relying on them for nutrients^80^.

The identification of *A. fumigatus* in the pristine culture was confirmed by recognising its colony’s morphological characteristics and isolating it in the fragment. Likewise, *S. pombe* was identified in the agar plates, swabs, and fragments while *A. oryzae* was detected in the fragments and swabs. Additionally, imaging has revealed the presence of *C. albicans*, *C. globosum*, *S. cerevisiae*, and *P. rubens,* which were also detected using nano-LC–MS analysis, affirming their well-established nature. These species, including *A. niger*, *A. terreus*, *F. oxysporum*, *Neurospora* spp*.,* and *Botrytis* spp*.,* are recognised allergens or are associated with human health issues^13,21,38,81,82^. *H. capsulatum*, *A. fumigatus,* and *C. albicans* are pathogens that can lead to mycoses with localised or progressive systemic infections^77^. Furthermore, some filamentous fungi have the ability to secrete mycotoxins, which are secondary metabolites that are toxic to vertebrates, into their surroundings^61,65^. Exposure to these toxins may result in acute and chronic health issues^14^, and can occur through inhaling toxigenic spores or direct skin contact. Aflatoxins, which are produced by *A. flavus,* are considered to be one of the most carcinogenic substances. Ochratoxin A is generated by many fungal species, including *A. niger*. Citrinin is produced by *Penicillium* and *Aspergillus* species*,* while Fumonisins are produced by several *Fusarium* species^77,82^. It is evident that the presence of biological species and cell debris has multiple implications, including the preservation of the artefacts. There are significant concerns regarding the health and safety of librarians, staff members, and visitors to museums and collection sites when handling archives, which need to be addressed^21,51^.

In conclusion, the study adopted a multi-scale approach to assess and describe the species richness of biodeteriorated seventeenth century Venetian historical handwritten letters. In this context, fluorescence microscopy was used for the first time to complement conventional imaging techniques. This methodology, unprecedented in the study of historical paper biodeterioration, allows the visualisation of biological species beyond and beneath the paper surface by means of their natural intrinsic autofluorescence and could provide a promising and effective tool for precisely locating microorganisms within the fibre network in a non-invasive and non-destructive way. Conventional culture-dependent and culture-independent proteomic analyses were used to unveil a rich community of microorganisms which discloses the coexistence of both fungal and bacterial species. Furthermore, this study provides evidence of a complex and thriving microbiota through the observation and detection of biological structures involving mite carcasses, insects, parasites, and possibly protists, for the first time. Previously unreported fungal, bacterial, and human pathogen species including *Histoplasma capsulatum*, *Brucella*, *Candida albicans* and species of *Aspergillus (A. flavus, A. fumigatus, A. oryzae, A. terreus, A. niger),* that cause infections or produce mycotoxins were documented for the first time in this study. These findings are crucial for the development of tailored preventive strategies and conservation treatments, which are necessary for the long-term preservation of paper-based historical archives. It is imperative to implement measures that limit progressive fragmentation to ensure longevity, but also guarantee the safety of personnel and users from potential health risks. Indicative approaches are periodic indoor airborne microbiological monitoring and material-specific cleaning processes, for contaminated items, that do not disrupt their weakened structure.

## Materials and methods

### Description of the materials and sampling

The Correr Museum Library, situated in Venice, Italy, houses a valuable collection of artworks, including manuscripts, letters, and printed volumes of great artistic and historical significance. This study analyses historical handwritten letters dating back to the seventeenth century preserved there. The letters belong to Italian Dandolo Family, renowned and distinguished in the history of Venice, and primarily concern political and diplomatic affairs. The letters were selected based on their deteriorated state, displaying pronounced aesthetic and structural alterations, observed on-site. While the Library has maintained optimal indoor conditions, the letters were not added to its collection until 1932. Prior to their acquisition, they were privately stored in a family residence under unknown storage conditions.

The initial in situ sampling approach involved a non-invasive method. Small pieces of adhesive tape were carefully applied over different areas of the surfaces of the paper sheets, focusing on the most damaged regions, to collect and retrieve accumulated debris. The tape pieces were then affixed to microscopy glass slides and kept in Falcon™ tubes. Sterile cotton swabs, used as received, were gently rubbed against these areas. Given the extensive material deconstruction of the letter sheets and the impossibility of restoration, sampling was deemed necessary to reveal the underlying factors contributing to their condition. In this minimally invasive process, minuscule fragments, approx. 10 mm in size, were collected with tweezers from the lower sections of the pages, where torn pieces were entirely detached, and placed in microtubes. These fragments were subsequently sectioned into smaller pieces in the laboratory to facilitate imaging, preparation of Petri dish cultures, and molecular digestion through culture-independent nano-LC–MS.

### Paper samples used as control

Whatman Grade 1 qualitative filter paper (standard grade cellulose filter paper, Cytiva) was used as a reference control to enable a comparison with the historical paper. Small pieces of the reference paper were cut and utilised to capture images of the fibre network at various magnifications using stereoscope, optical and scanning electron microscopy. Additionally, small pieces of filter paper were used as a control for inoculating the cultures in agar Petri dishes and conducting the culture-independent experiment using nano-LC–MS digestion. Sterile cotton swabs, used as received, were also delicately rubbed against these surfaces, and their tips were analysed.

### Microscopic analysis

The fragments were preliminary examined using a Nikon SMZ745T stereoscopic microscope, with backwards illumination, coupled to a CCD camera. Image acquisition was realised with the use of the Alexasoft X-entry software. Selected ROI were visualised with an Olympus BX43 optical microscope, coupled to a CCD camera. Image acquisition was realised through the CellSens entry software. Fluorescence microscopy imaging was conducted using an optical microscope, equipped with a white light fluorescence illumination system (CoolLed pe-300 white series) with individual control of three excitation channels covering the UV–Vis, blue and green–yellow–red regions. Microscopic analysis was conducted in room temperature conditions, without any prior sample preparation. Microscopy glass slides covered with pieces of adhesive tape were directly examined, as prepared in situ. Control samples (Whatman Grade 1 qualitative filter paper) were likewise observed using stereoscopic and optical microscope, employing different magnification levels. Optical microscopy images obtained in both brightfield and fluorescence modes were analysed using an open source scientific image processing program (ImageJ)^83^.

### SEM analysis

A high-performance scanning electron microscope (SEM JEOL 6490LA) was used to perform the analysis of the paper fragments from the perspective of morphological features of microorganisms and interactions with the fibre network. The instrument was operated at an acceleration voltage of 10–20 kV, and the paper fragments were coated with gold before being placed onto the sample holders. The images were captured in secondary electron (SE) mode with high resolution under a magnification range of 500–5000×. Control samples (Whatman Grade 1 qualitative filter paper) were also visualised under a magnification range of 300–3000×.

### Culture-dependent analysis

Four small paper pieces (#1, #2, #3, #4) weighing approx. 10 mg were used for the isolation of the different biological species. The fragments were analysed in triplicates by submerging them in Petri dishes containing three different media to promote growth irrespective of the strains: (i) PCA (Plate Count Agar, Millipore, Merck group, Milan, Italy), (ii) MEA (Malt Extract Agar, Microbiol, Cagliari, Italy) supplemented with 15% NaCl (sodium chloride, bacteriological, Microbiol), and (iii) DG18 (Dichloran Glycerol agar base, Microbiol, Cagliari, Italy) supplemented with chloramphenicol antibiotic. The inoculations were conducted in a sterile environment within a laminar flow hood. Small pieces of Whatman filter paper were also inoculated in parallel as a reference control (sample #0). The Petri dishes were incubated at 20 °C in a refrigerated thermostat incubator (Velp Scientifica FOC 225E). Daily monitoring facilitated the timely detection of the growth of different fungal and bacterial species. Micro-colonies were isolated from the primary cultures using adhesive Fungi tape™ (Scientific Device Laboratory) specifically designed for mycology slide preparation, and glass slides were visualised with an optical microscope (Axioplan, Axiocam ERc5S, Zeiss Germany, Zen blu lite 2011 software). Re-inoculations into fresh media were prepared for further analysis. Images of the Petri dishes were captured to record the growth evolution of the species over time.

### Culture-independent analysis with nano-LC–MS

The samples comprised four small paper fragments (#1, #2, #3, #4) weighing approx. 10 mg, sterile cotton swabs, and agar plate colonies. Small pieces of Whatman filter paper, as well as sterile cotton swabs rubbed on Whatman filter paper, were used as a control reference (blank samples). The paper fragments and the cotton tips of the swabs were dipped in a microtube containing 200 µL of reducing solution (Dithiothreitol at 10 mM) in digestion buffer and incubated at 56 °C for 1 h. The digestion buffer consisted of 50 mM ammonium bicarbonate in Milli-Q water (pH 8). Then, 200 µL of alkylating solution (Iodoacetamide at 20 mM) in digestion buffer were added in the same microtube and incubated at RT for 1 h in the dark. All chemicals were purchased from Merck Life Sciences (Milan, Italy). At the end of the incubation time, 100 µL of digestion buffer were added in the microtube together with 2 µL of trypsin solution and the samples were incubated at 37 °C overnight at 600 rpm. Trypsin (proteomics grade) was dissolved to a final concentration of 1.2 µg µL^−1^ in H_2_O + 0.1% formic acid before use. The following day, the supernatant was recovered, and the peptides were desalted and concentrated using desalting Pierce C18 Spin Columns (Thermo Fisher Scientific, Rockford, IL, USA), following the protocol recommended by the vendor. The resulting peptides were then analysed by high-resolution LC–MS on Orbitrap Exploris 480 mass spectrometer (Thermo Scientific) equipped with a nanoelectrospray source and coupled with a nano-LC system. The peptides were eluted with a linear gradient of acetonitrile in water (3 to 50%). Both eluents were added with 0.1% formic acid. Data were acquired in data-dependent mode, selecting multiply charged stated (2 + to 6 +) as precursors for MS/MS fragmentation. The resulting raw spectra were searched against the bacteria, fungi, and yeast proteomes, using Proteome Discoverer software (Thermo Scientific). These corresponding FASTA files were downloaded from UNIPROT, a freely accessible database of protein sequence. Only reviewed entries were used for the data search. A putative fungal protein sequence was considered a positive hit under 1% maximum false discovery rate (FDR), and if at least two peptides were positively assigned to a unique protein hit, as done in similar studies^26,27,84^. Blank samples were used as procedure control reference, i.e., treated, and acquired as described above. Protein hits observed in the blanks were excluded from the protein hits observed in the samples, being considered as background noise for the experiment. Similar procedure was followed for the analysis of the agar plate colonies. Information on the species was also extracted from GenBank, an open access genetic database of nucleotide sequences and their protein translations of NIH (National Center for Biotechnology Information), part of the International Nucleotide Sequence Database Collaboration.

### Supplementary Information

Supplementary Figures.

## Data Availability

The datasets generated during and/or analysed during the current study are available from the corresponding author on reasonable request.
