# Supplementary material for: Fungal and bacterial species richness in biodeteriorated seventeenth century Venetian manuscripts
Source: Sci Rep. 2024 Mar 25;14:7003. doi: 10.1038/s41598-024-57228-2 (PMC10961312; doi:10.1038/s41598-024-57228-2)

**Supporting Information**

# Title

Fungal and bacterial species richness in biodeteriorated 17^th^ century Venetian manuscripts

# Authors

Maria Stratigaki,^a*^ Andrea Armirotti,^b^ Giuliana Ottonello,^b^ Sabrina Manente,^c^ Arianna Traviglia^a^

# Author affiliations

^a^ Center for Cultural Heritage Technology (CCHT), Istituto Italiano di Tecnologia, Via Torino 155, 30172 Venice, Italy

^b^ Analytical Chemistry Facility, Istituto Italiano di Tecnologia, Via Morego 30, 16163 Genova, Italy

^c^ Department of Molecular Sciences and Nanosystems, Ca’ Foscari University of Venice, Via Torino 155, 30172 Venice, Italy

*Corresponding author, [maria.stratigaki@iit.it](mailto:maria.stratigaki@iit.it)

Fig. SI_1 Images of the fibre network of Whatman Grade 1 filter paper at different magnifications captured with: (a) stereoscope, scale bar 500 µm, (b) optical microscope, scale bar 100 µm, (c) scanning electron microscope, scale bars as denoted individually.


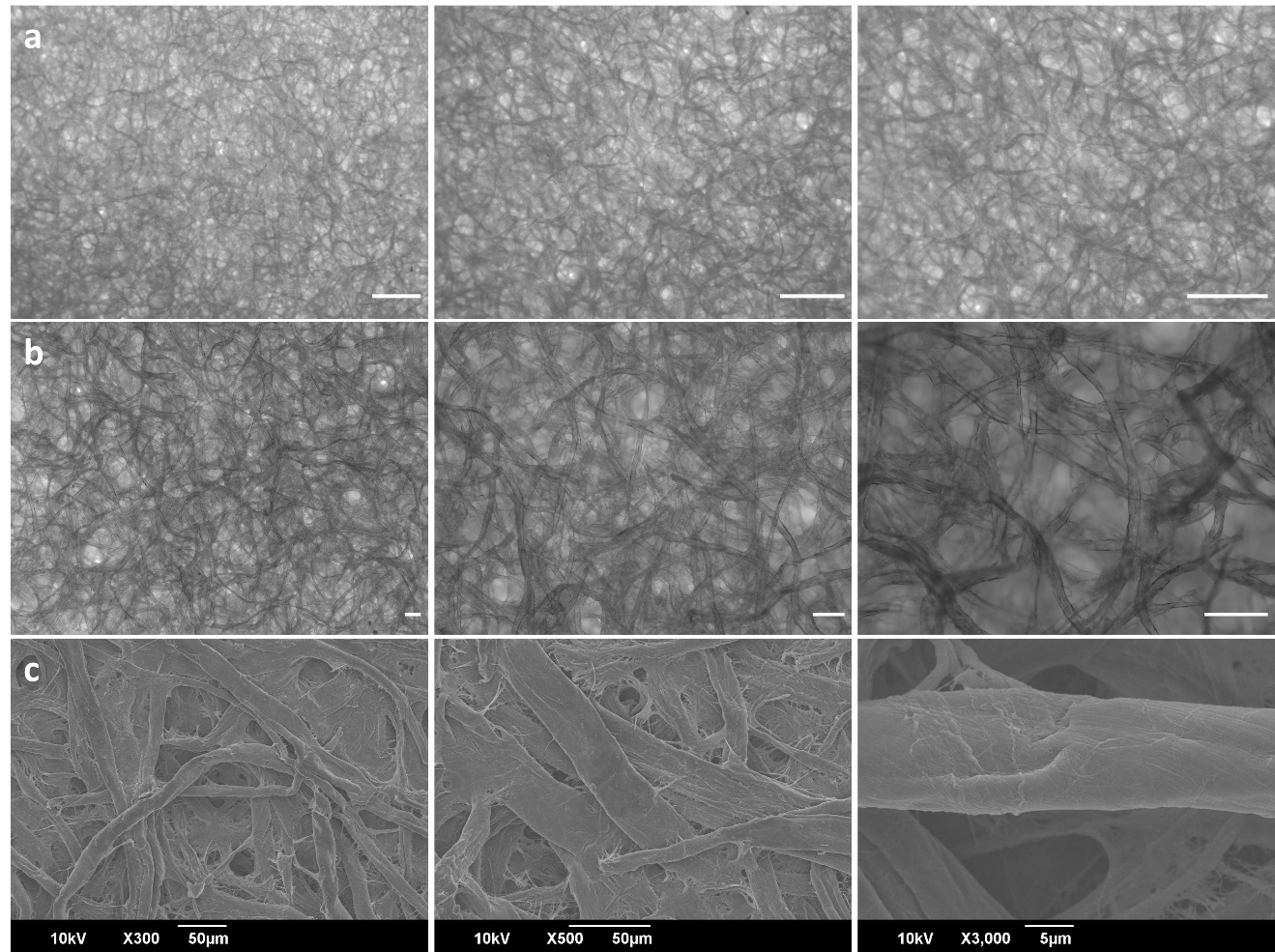


Fig. SI_2 (a) SEM image of fragment #3, scale bar 50 µm, and (b) stereoscopic view of a golden-brown coloured parasite of approximate length of 3 mm and width 1 mm, discovered between the letters, scale bar 500 µm.


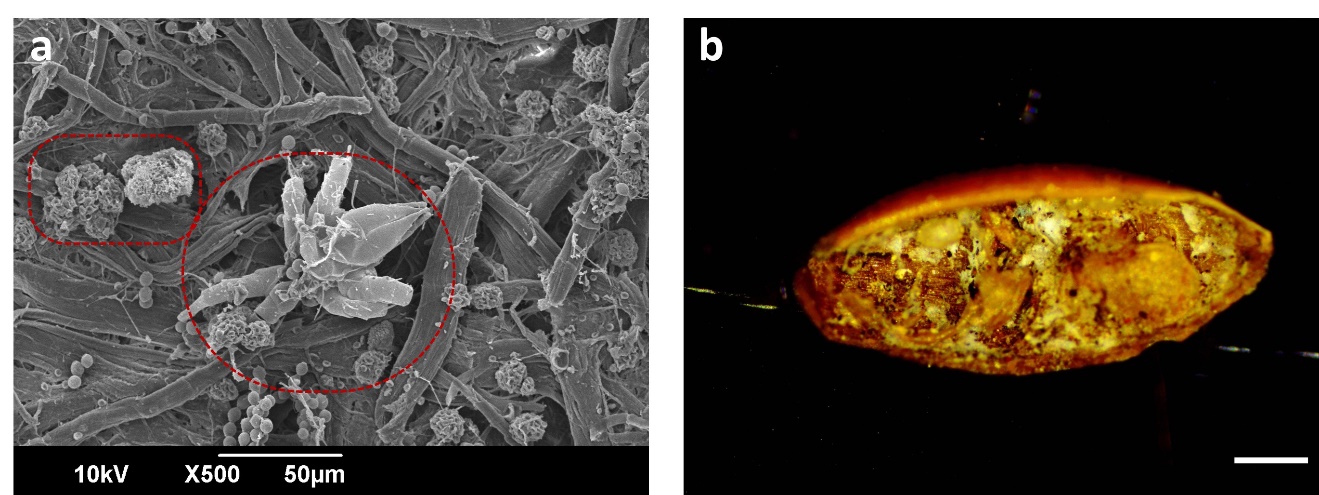


Fig. SI_3 Images of Petri dishes showing the growth evolution of microorganisms after inoculation of the fragments on agar plates captured at different days: (i) day 1, (ii) day 8, (iii) day 11, (iv) day 15, (v) day 24, and (vi) day 30. Media are: (a) PCA, (b) DG18, and (c) MEA.


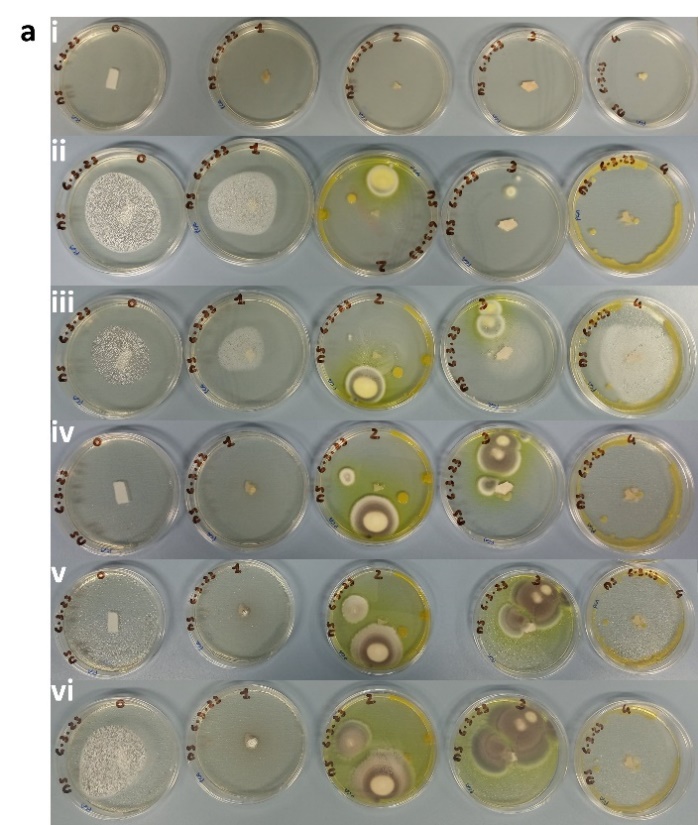

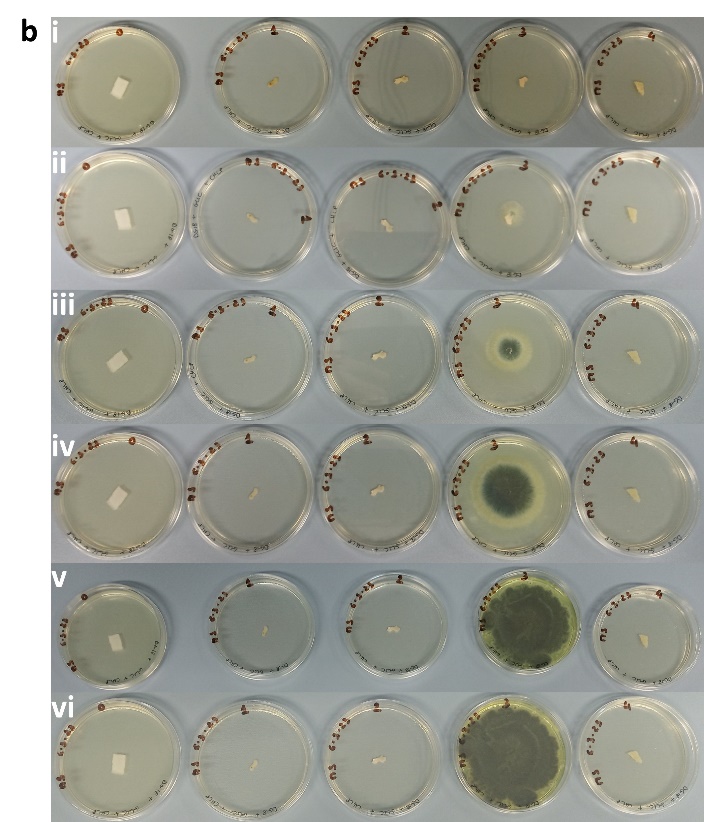

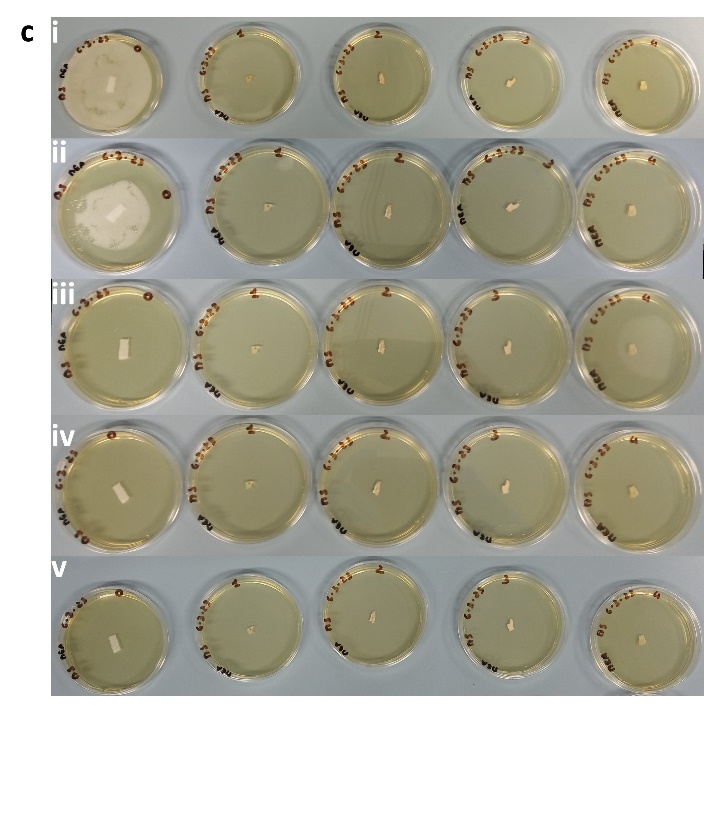


Fig. SI_4 Images of Petri dishes showing re-cultures after separating the different species from the original colonies: (a) sample 1-PCA on two new plates of PCA (left) and MEA (right) media, day 21, (b) sample 2-PCA on three new plates of PCA media, day 7, (c) sample 4-PCA on four new plates of PCA media, day 7.


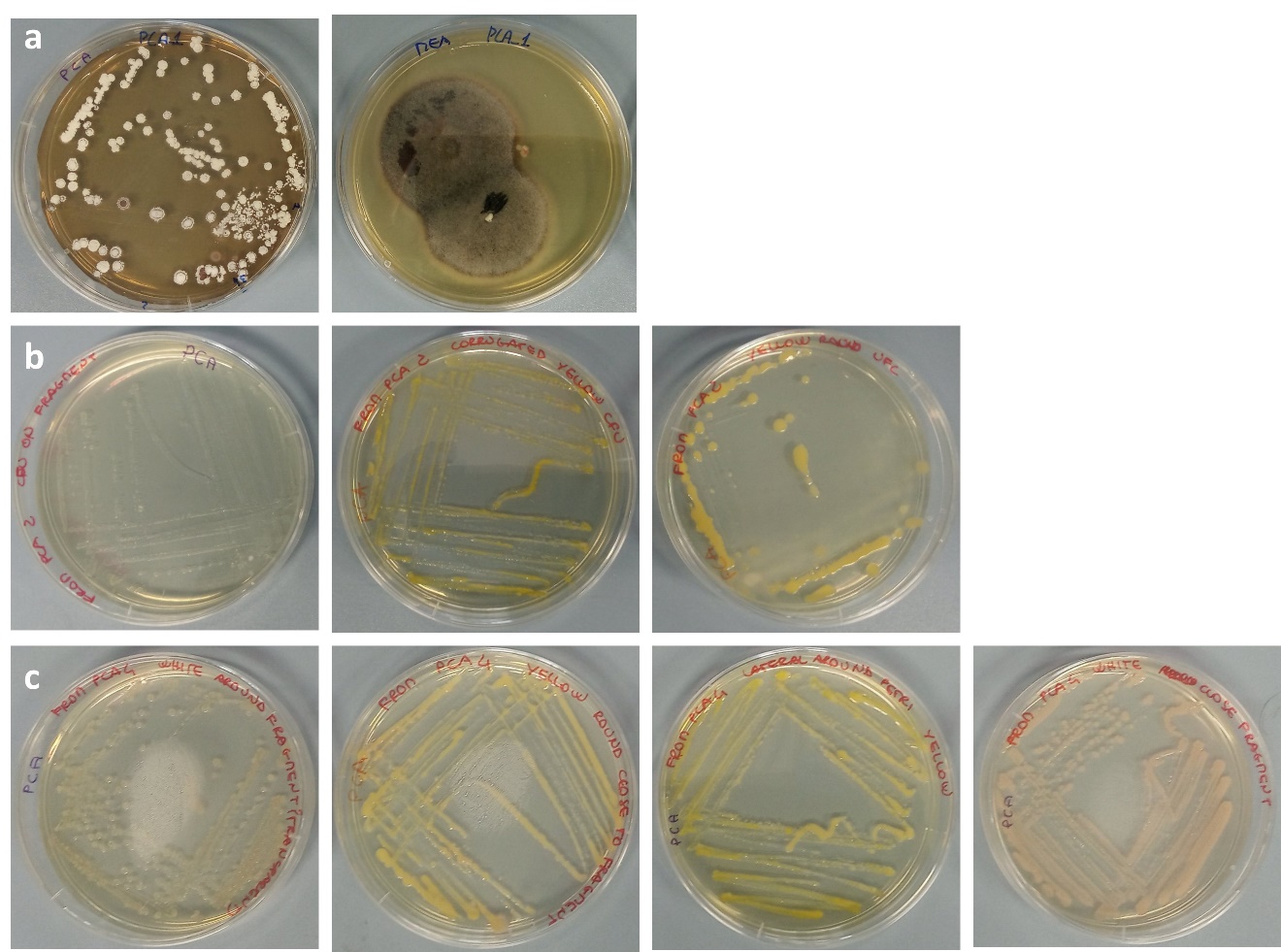

Supplement: Supplementary file 1 — Supplementary Figures. [file 41598_2024_57228_MOESM1_ESM.docx]
